# Effect of esketamine combined with dexmedetomidine on delirium in sedation for mechanically ventilated ICU patients: protocol for a nested substudy within a randomized controlled trial

**DOI:** 10.1186/s13063-024-08287-3

**Published:** 2024-07-02

**Authors:** Wenhui Zhang, Jinjin You, Jing Hu, Xiangding Chen, Han Wang, Nan Li, Chen Wei, Wanchun Tang, Xiangrong Zuo

**Affiliations:** 1 Department of Critical Care Medicine, The First Affiliated Hospital of Nanjing Medical University, Nanjing, Jiangsu People’s Republic of China; 2 Department of Pharmacy, The First Affiliated Hospital of Nanjing Medical University, Nanjing, Jiangsu People’s Republic of China

**Keywords:** Esketamine, Dexmedetomidine, Delirium, Respiration, Artificial, Intensive care units, Randomized controlled trial

## Abstract

**Background:**

Use of sedatives and analgesics is associated with the occurrence of delirium in critically ill patients receiving mechanical ventilation. Dexmedetomidine reduces the occurrence of delirium but may cause hypotension, bradycardia, and insufficient sedation. This substudy aims to determine whether the combination of esketamine with dexmedetomidine can reduce the side effects and risk of delirium than dexmedetomidine alone in mechanically ventilated patients.

**Methods:**

This single-center, randomized, active-controlled, superiority trial will be conducted at The First Affiliated Hospital of Nanjing Medical University. A total of 134 mechanically ventilated patients will be recruited and randomized to receive either dexmedetomidine alone or esketamine combined with dexmedetomidine, until extubation or for a maximum of 14 days. The primary outcome is the occurrence of delirium, while the second outcomes include the number of delirium-free days; subtype, severity, and duration of delirium; time to first onset of delirium; total dose of vasopressors and antipsychotics; duration of mechanical ventilation; ICU and hospital length of stay (LOS); accidental extubation, re-intubation, re-admission; and mortality in the ICU at 14 and 28 days.

**Discussion:**

There is an urgent need for a new combination regimen of dexmedetomidine due to its evident side effects. The combination of esketamine and dexmedetomidine has been applied throughout the perioperative period. However, there is still a lack of evidence on the effects of this regimen on delirium in mechanically ventilated ICU patients. This substudy will evaluate the effects of the combination of esketamine and dexmedetomidine in reducing the risk of delirium for mechanically ventilated patients in ICU, thus providing evidence of this combination to improve the short-term prognosis. The study protocol has obtained approval from the Medical Ethics Committee (ID: 2022-SR-450).

**Trial registration:**

ClinicalTrials.gov: NCT05466708, registered on 20 July 2022.

**Supplementary Information:**

The online version contains supplementary material available at 10.1186/s13063-024-08287-3.

## Background {6, 7 and 8}

Delirium, a neuropsychiatric syndrome, is characterized by an acute onset and fluctuation in attention, awareness, and cognition and often triggered by multiple predisposing factors (e.g., advanced age or alcohol use) and precipitating factors (e.g., acute medical illness, trauma, or major surgeries) [1]. The interaction between multiple internal and external factors renders ICU patients highly susceptible to delirium. It is reported that delirium occurs in 31.4~73.0% of ICU patients [2, 3]. Mechanical ventilation (MV) in the ICU has been proven as an independent risk factor for delirium [4], which can, in turn, induce the prolongation of MV and hospital length of stay (LOS), re-admission, and even long-term cognitive impairment [5]. Ely et al. have reported that during the ICU stay, as many as 81.7% of patients on ventilators develop delirium, a strong independent predictor of mortality [6]. Despite these detrimental effects of delirium, there still lack of evidence about what medications can prevent delirium.

For patients undergoing MV, sedatives and analgesic are routinely administered to synchronize their mechanical breaths, increase tolerance to other invasive procedures, decrease systemic metabolism, and protect organ reserve functions. However, these medications, especially when overdosed, pose a risk for delirium [7, 8]. Benzodiazepines, the most commonly prescribed sedatives in the ICU, tend to accumulate in adipose tissues, particularly in the elderly patients and those with impaired kidney function. Long-term infusion of benzodiazepines may delay recovery and extubation in MV patients, thus bringing with a high risk of delirium. Riker et al. have reported that the incidence of delirium reaches 76.6% in patients sedated with midazolam and 55.0% in those who are CAM-ICU negative at baseline [9]. Therefore, opioid-sparing and nonbenzodiazepine sedatives are expected to reduce delirium risk in MV patients.

Dexmedetomidine, a nonbenzodiazepine, has been recommended by the PADIS guideline since 2013 [10]. A meta-analysis provides evidence that dexmedetomidine can significantly reduce the risk of delirium (Relative Risk, 0.67), duration of MV, and LOS in the ICU [11]. Even in the PRODEX trial, dexmedetomidine is accompanied by a lower rate of neurocognitive adverse events (18.3% versus 28.7%, *P *= 0.008) and less treatment for these adverse events (15.0% versus 24.7%, *P* = 0.009) at 48 h after sedation cessation, compared to propofol, another common nonbenzodiazepine [12]. Additionally, dexmedetomidine exerts an adjunctive analgesic effect, thus diminishing the need for opioids and further the risk of opioid-related delirium.

However, dexmedetomidine also elicits some undesirable effects. First, it may increase the risk of hypotension and bradycardia [11], especially in critical conditions, such as septic shock or post-major surgery, in which patients’ hemodynamics is unstable. Second, the sedative effects of dexmedetomidine vary significantly among patients and fail in approximately 9~14% of patients [12], thus increasing the risk of self-extubation [11]. Therefore, a combination regimen may mitigate the adverse effects of dexmedetomidine and maximize its prophylactic effects against delirium.

Ketamine or its enantiomers can be combined with dexmedetomidine in anesthesia [13] and magnetic resonance imaging [14] as well as analgosedation for invasive procedures (e.g., burn wound dressing) [15]. Ketamine can shorten the time for sedatives to act and counteract the side effects induced by dexmedetomidine, such as hypotension and bradycardia [14, 15]. Conversely, dexmedetomidine can attenuate the side effects of ketamine on patients’ psychology [15]. The ketamine-dexmedetomidine combination provides satisfactory sedative and analgesic effects, hemodynamic stability, and milder respiratory depression [16]. Given the high incidence of shock in MV patients, other agents are suggested to combine with dexmedetomidine to maximize its efficacy and safety in reducing the risk of delirium, shortening ICU LOS, decreasing mortality, and improving short-term prognosis in MV patients. Esketamine, a dextrorotatory enantiomer of ketamine, presents a shorter elimination time and sedative and analgesic effects at least twice as potent as those of racemic ketamine. Accordingly, esketamine causes fewer neurocognitive adverse reactions [17]. Esketamine fulfills its neuroprotective effect by blocking the N-methyl-d-aspartate (NMDA) receptors to prevent neurons from apoptosis and antidepressant effects by decreasing inducible nitric oxide synthases [18]. Esketamine also enhances brain function in critically ill patients, thus lowering the risk of delirium. In general anesthesia, esketamine can reduce the incidence of postoperative delirium and quicken neurocognitive recovery in elderly patients [19, 20]. Administered at the end of anesthesia, esketamine can also reduce the incidence of emergence delirium in preschool children [21]. Nevertheless, clinical trials on the efficacy and safety of esketamine-dexmedetomidine combination remain to be expanded.

In our previous trial, Safety and Efficacy of Esketamine combined with Dexmedetomidine for Sedation of mechanically ventilated patients in the ICU: a prospective, randomized, single-center study (SEEDS trial), preliminary but unpublished results have shown a 20.83% reduction in the incidence of delirium in MV patients sedated with the combination of esketamine with dexmedetomidine, as compared with dexmedetomidine alone. This efficacy should be warranted in further research. Here, we design this randomized, active-controlled, 1:1 allocation trial, which will be nested in the main trial. This substudy aims to evaluate the effect of dexmedetomidine-esketamine combination vs dexmedetomidine alone on delirium for prolonged sedation in critically ill MV patients and the benefits of this combination regimen on short-term prognosis.

## Methods

### Study design and setting {9}

This substudy is a single-center, randomized, active-controlled, superiority trial nested in the SEEDS main trial (ClinicalTrials.gov NCT05466708). Our SEEDS trial aims to evaluate the safety and efficacy of esketamine-dexmedetomidine combination for prolonged and light sedation of MV patients in the ICU. The benefits and risks of this regimen will be examined in critically ill patients admitted to the general ICU (32 beds) of The First Affiliated Hospital of Nanjing Medical University, a tertiary academic hospital in China. This substudy will be conducted over a period from August 2023 to December 2024. The protocol will be presented according to the Standard Protocol Items: Recommendations for Interventional Trials (SPIRIT) (see Additional file 1 for SPIRIT checklist).

## Eligibility criteria {10}

### Population

In both the SEEDS main trial and substudy, critically ill patients admitted to ICUs and given MV with oral endotracheal intubation will be recruited. Included will be those who are (a) transferred to the ICU after oral endotracheal intubation in the emergency room or general wards and ventilated for no more than 24 h, (b) admitted to the ICU with endotracheal intubation after surgery, and (c) indicated to get emergent intubation in the ICU. The inclusion and exclusion criteria are designed as follows.

### Inclusion criteria

Patients who meet all the following criteria are eligible for the substudy.

- Mechanically ventilated with oral endotracheal intubation in the ICU
- Expected to spend more than 24 h on ventilators
- At an age of 18~70 years

### Exclusion criteria

- Known or suspected allergy to esketamine, dexmedetomidine, remifentanil, or propofol
- Intraoperative use of esketamine
- Acute myocardial infarction, left ventricular ejection fraction less than 30%, or third-degree atrioventricular block contraindicative of dexmedetomidine
- Seriously high intracranial pressure, hyperthyroidism, or refractory hypertension contraindicative of esketamine
- Pregnancy or lactation period
- Obesity (defined as body mass index > 35 kg/m^2^)
- Severe hemodynamic or respiratory instability due to severe burn or trauma (defined as Injury Severity Score ≥ 25)
- Heavy drinking defined by the WHO as drinking at least 60 g of pure alcohol every day for men and at least 40 g for women
- Terminally ill patients near death, such as patients with extensively metastatic tumor or refractory shock
- Acute or chronic severe liver disease (Child-Pugh class C or history of liver transplantation)
- Acute or chronic renal insufficiency needing dialysis
- Long-term exposure to sedatives, opioid analgesics, or antianxiety drugs
- Severe central nervous system disease such as cerebrovascular accidents, status epilepsy, or post cardiac arrest indicative of hypothermia
- Continuous deep sedation, defined as the Richmond Agitation and Sedation Scale (RASS) − 4~− 5
- Patients or authorized surrogates refuse to provide informed consents or withdraw consents within the first 24 h after administration of sedatives
- Mechanically ventilated for more than 24 h prior to enrollment (not including the time on ventilators in the operation room)
- History of dementia, Alzheimer’s disease, schizophrenia, or other mental diseases
- History of delirium
- Inability to co-operate with the Confusion Assessment Method for ICU (CAM-ICU) due to neurological problems and visual or auditory impairments

### Dropout criteria

- Patients whose condition continue to deteriorate to the requirement for deep sedation or neuromuscular blockers
- Patients whose renal function deteriorates to the requirement for dialysis
- Patients whose consciousness deteriorates due to acute cerebrovascular stroke
- Severe adverse events
- Patients or authorized surrogates decide to withdraw from the study
- The reason for dropout will be elaborated in the case report forms (CRFs)

## Interventions

### Intervention-details {11a}

After randomization, previous analgesics and sedatives will be discontinued. Participants are required to stay in a RASS range of − 2~0 before initiation of study drugs. Both arms are first subjected to analgesia with remifentanil. The dose of remifentanil starts from 0.02 μg/kg/min and increases by 0.025 μg/kg/min every ≥ 5 min until the Critical-care Pain Observation Tool (CPOT) reaches 0. The infusion rate of remifentanil can be increased temporarily at 5 min prior to invasive procedures. The study drugs will be dispensed at the concentrations shown in Table 1 by nurses who are not involved in the study. In the combination arm, the loading dose is 1 mg/kg for esketamine and 1 μg/kg for dexmedetomidine. After that, esketamine will be initially dosed at 0.25 mg/kg/h and increased by 0.125 mg/kg/h every 15 min. Meanwhile, dexmedetomidine will be initially dosed at 0.2 μg/kg/h and increased by 0.1 μg/kg/h every hour, to achieve a RASS range of − 2~0. In the dexmedetomidine arm, dexmedetomidine is the only sedative tittered in the same to achieve the same RASS range. For participants who fail to be sedated, rescue propofol will be allowed at the lowest dose. Any use of benzodiazepines and other opioids is discouraged (if emergence agitation occurs and propofol is difficult to be controlled, a 5-mg bolus of midazolam can be administrated intravenously or intramuscularly). Study drugs will be infused until extubation or 14 days maximum. Participants spending more than 14 days on ventilators will be sedated according to standard care.

**Table 1:** Administrative protocol

Study drugs	Manufacturer	Route	Concentration	Volume	Loading dose	Maintain dose
Remifentanil	Yichang Humanwell	Syringe pump	80 µg/mL	50 mL	None	0.02~0.15 μg/kg/min^b^
Esketamine	Jiangsu Hengrui	Syringe pump	6 mg/mL	50 mL	1 mg/kg	0.25~1.5 mg/kg/h^b^
Dexmedetomidine	Jiangsu Hengrui	Syringe pump	8 µg/mL	50 mL	1 μg/kg^a^	0.2~0.7 µg/kg/h^b^
Propofol	Corden Pharma	Syringe pump	10 mg/mL	20 mL	None	1~4 mg/kg/h

^a^Intravenous injection over 20 min

^b^Administration over the maximal dose is not allowed

### Intervention-modification {11b}

The sedative status of participants will be assessed using RASS every 4 h by investigators. Participants who are insufficiently sedated by study drug, though at the maximum dose, can receive a rescue propofol bolus dose of 25~50 mg; then, a continuous intravenous infusion according to the protocol in Table 1. When oversedation occurs, considering the hypnotic effect of remifentanil and the analgesic effect of esketamine and dexmedetomidine, the dose of remifentanil can be reduced first as long as the CPOT score maintains at 0. Then, study drugs are infused at a lower rate or discontinued if the RASS score fails to respond. For participants who appear to have respiratory depression, coma, and myosis induced by overdose of remifentanil, the infusion rate should be decreased immediately, and naloxone or nalmefene can be administrated at the researcher’s discretion. If one participant’s heart rate decreases by more than 50% from that at baseline, or to less than 50 beats per minute, dexmedetomidine will be discontinued. After the heart rate returns to the baseline level, dexmedetomidine and isoprenaline can be administrated concomitantly.

### Intervention-adherence {11c}

Study drugs may induce adverse events. The sympatholytic effect of dexmedetomidine and remifentanil and the sympathomimetic effect of esketamine are likely to cause hemodynamic instability. Fluids and/or vasoactive agents can be administered by the clinical team to enhance participants’ adherence to the protocol. Other strategies include the following: (a) assigning an investigator to prescribe study drugs and supervise the administration and (b) writing the titration procedure and administrative protocol of study drugs on whiteboards beside beds, to remind the participant not to violate the protocol.

### Intervention-concomitant care {11d}

The efforts to prevent delirium in both arms consist of treating primary diseases, minimizing light and noise, optimizing sleep, early mobility, family engagement, etc. Olanzapine or other antipsychotics will be prescribed to treat delirium for participants in both arms who show no QT interval prolongation or severe arrhythmia. Sedatives will be withdrawn and extubation will be performed in both arms following the standardized protocol. Participants will be assessed for whether they can tolerate sedation interruption every morning. An answer of “Yes” to any of the following items means that sedatives are imperative for them and they are not eligible for sedation interruption: severe hypoxemia, myocardial ischemia, hypertensive crisis, status asthmaticus, escalating sedative doses due to ongoing agitation, and use of neuromuscular blockers. Otherwise, the infusion of study drugs will be discontinued by the investigator at 9:00 AM. Remifentanil is infused into patients after surgeries. During the interruption of sedation, if participants exhibit severe dyspnea, SpO_2_ < 88% lasting over 5 min, or new onset of arrhythmia, the sedatives will be restarted at half of the previous dose, and then the investigator will re-titrate sedatives till into the target RASS range. Awakening is defined as an ability to perform at least 3 of the following movements on request: eye opening, tracking, hand squeezing, and toe moving. The eligible participants will be screened out for the spontaneous breath trial (SBT) (see Table 2 for protocol of SBT). When participants pass the SBT, extubation will be decided by experienced clinicians.

**Table 2:** Preparedness test and failure criteria for SBT

Preparedness test for SBT^a^	Failure criteria of SBT^b^
Causes leading to respiratory failure were eliminated	Respiratory rate > 35 or < 8 breaths per minute
FiO_2_ ≤ 50% and PEEP ≤ 5 cm of water	SpO2 < 90%
pH > 7.35 and PaO_2_/FiO_2_ > 200 mmHg	Depressed mental status or agitation
Heart rate < 100 beats per minute	Hemodynamic instability
Without or with minimal doses of vasoactive agents	Heart rate > 140 or < 60 beats per minute or new onset of arrhythmia
Temperature < 38 °C	Severe dyspnea defined as respiratory rate/tidal volume > 105
Hemoglobin > 90 g/L	Tidal volume < 4 mL/kg when spontaneous breathing
Adequate cough	Increased accessory-muscle activity

^a^Participants who respond with “Yes” to all of the following items are eligible for SBT

^b^SBT will be performed using continuous positive airway pressure with a pressure-support level of 7 cm of water for half an hour. An answer of “Yes” to any of the following items suggests an immediate cessation of SBT. SBT will be performed daily until successful weaning

### Provisions for post-trial care {30}

Sedatives and analgesics needed after extubation will be prescribed according to standard care.

## Outcomes {12}

### Primary outcome

The primary outcome of this substudy is set as the occurrence of delirium, because that a lower occurrence of delirium is a factor that intensivists depend on to select sedatives for MV patients. Delirium is measured by the Confusion Assessment Method for ICU (CAM-ICU) which covers four indexes: (1) acute onset of mental status change or fluctuation of mental status, (2) inattention, (3) altered consciousness, and (4) disorganized thinking. The CAM-ICU is considered to be positive with the presences of indexes 1 plus 2 plus 3 or 4. The Chinese version of CAM-ICU has been validated with sensitivities of 91.8~93.4% and specificities of 87.7~90.8% [22]. Participants will be assessed twice a day (08:00~10:00 and 16:00~18:00, respectively) by two trained and blinded investigators independently from enrollment (day 0) to day 14 or discharge from the ICU or dropout, whichever comes first. If there is a disagreement, a third senior investigator will recheck the results. Acute change in mental status observed beyond the above time period will be immediately evaluated. The incidence of delirium is defined as the proportion of participants who have at least one positive CAM-ICU.

### Secondary outcomes

The secondary outcomes are categorized into delirium-related and prognosis-related. Delirium-related outcomes will help us to explore the potential mechanism contributing to the difference between two arms.

- The number of delirium-free days (the number of days without a positive CAM-ICU) until 14 days after enrollment or discharge from the ICU
- Subtype of delirium: hyperactive, hypoactive, or mixed
- Severity of delirium: the severity will be assessed using a four-item version of CAM-S score [23], with the highest score being 7. Similar to the primary outcome, the CAM-S score will be independently evaluated by two blinded investigators twice a day, and a senior clinician will be invited to resolve the disagreement
- Time to the first onset of delirium: the number of hours from enrollment to the first positive CAM-ICU
- Duration of delirium: after the day of onset, the number of days on which participants have a positive CAM-ICU until 14 days after enrollment or ICU discharge. Delirium is ceased after 12 consecutive hours of no recurrence since its first resolution
- Total dose of vasopressors and antipsychotics

Prognosis-related outcomes:

- Duration of MV: the number of hours from intubation to extubation, not including the time spent in the operating room
- ICU LOS: ICU LOS is defined as the number of days from ICU admission to ICU discharge without re-transfer to ICU within 24 h
- Accidental extubation, re-intubation, and re-admission: accidental extubation is defined as premature removal of the endotracheal tube by the patient; re-intubation is defined as requirement for invasive mechanical ventilation within 48 h after extubation; re-admission is defined as re-transfer to ICU after 24 h)
- In-ICU mortality, mortality at 14 and 28 days

### Participant timeline {13}

The participant timeline is shown in Table 3.

**Table 3:**
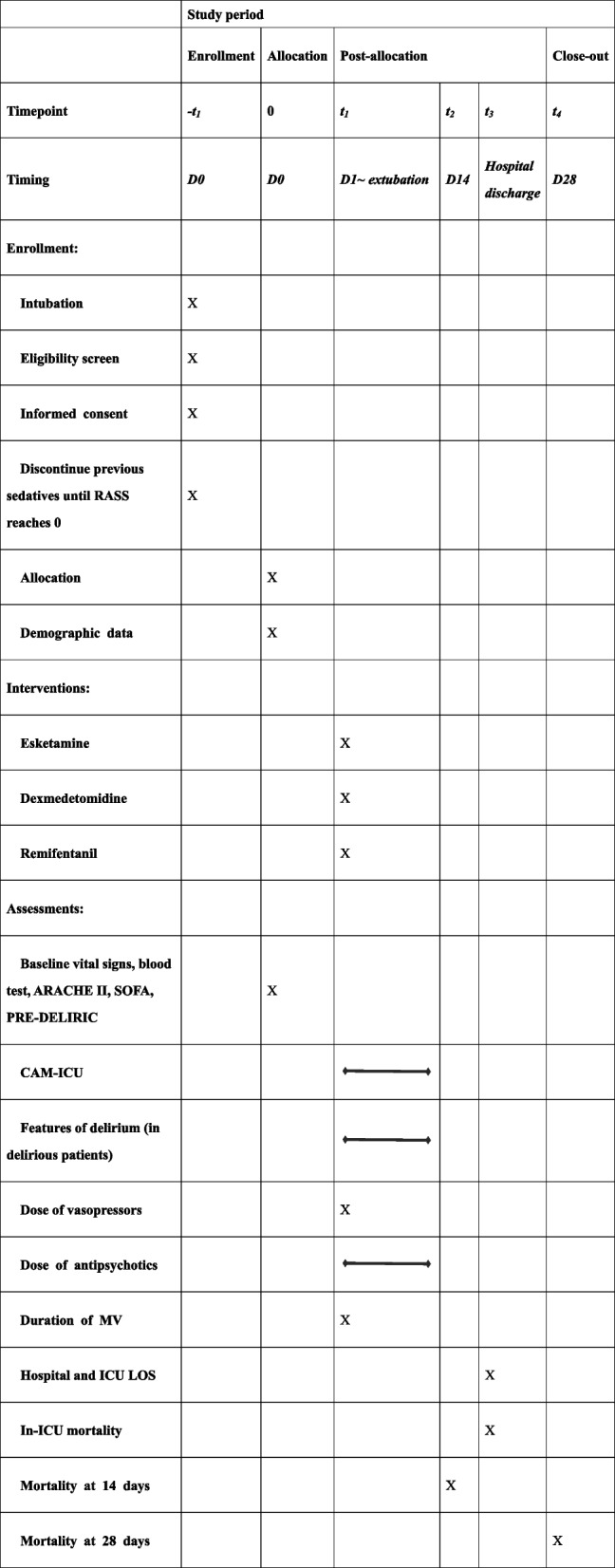
Flowchart of enrollment, interventions and assessments

### Sample size {14}

The sample size is estimated based on the incidence of delirium. Data are lacking about comparing two sedation regimens in critically ill patients. We therefore conducted a meta-analysis, and the results showed that the incidence of delirium in the esketamine group was lower than that in the control group (Odds Ratio 0.26, 95% confidence interval 0.12~0.56, *P* = 0.0007). Based on the results of the meta-analysis and previous studies, we assume that the incidence of delirium in MV patients sedated with dexmedetomidine is 30%, and the odds ratio is 0.26. A total of 60 participants will be needed for each arm at a significance level of 0.05 (two-tailed) and a power of 80%. It is expected that 10% of participants will withdraw from the study, as indicated in our SEEDS main trial. Therefore, we increase the sample size to 67 per arm, resulting in a total of 134 participants. The trial by Lu et al. [24] has shown a reduction of approximately 30% (from 40.00 to 10.30%) in the incidence of emergence delirium among participants receiving the combination regimen to induce anesthesia. A sample size of 67 in each arm achieves a 98.8% power to detect such a difference. Sample estimation is based on the Chi-Square Test (Unpooled) by PASS 15 (NCSS, LLC. Kaysville, Utah, USA).

### Recruitment {15}

Before recruitment, we will first invite clinicians in common wards and emergency room to report us the information about the patients who need emergency intubation and ICU referral. In the operating room, surgeons will collect the information of patients undergoing major surgeries before transferring them to the ICU. Investigators in our team will go through medical records to determine whether they are eligible for our study. Participants who have indications for intubation in our ICU are also recruited. After providing their informed consent, these participants are enrolled and randomized to two arms.

## Allocation

### Sequence generation {16a}

Eligible participants will be randomly assigned to two arms in a ratio of 1:1. A computer generated randomization sequence, with a fixed block, will be completed by principal investigator (PI) using SPSS 27.0 (IBM, USA). The incidence and severity of delirium may change with time and environment in the ICU. Block randomization will be used to reduce such periodic imbalances.

### Concealment mechanism {16b}

In case other investigators deduce the allocation sequence, the block size, the seed number, and the sequence are generated and concealed by PI, using sequentially numbered and opaque envelopes. The envelopes should only be opened when authorized surrogates have signed informed consent and sedatives are about to be prescribed.

### Implementation {16c}

Eligible participants will be screened out and enrolled by one investigator You, according to the inclusion and exclusion criteria. After confirming the participation, investigator Zhang will take the envelopes from PI and then prescribe the medications according to the study protocol. Hospital pharmacists will assign the drugs directly to bedside nurses.

## Blinding

### Who will be blinded {17a}

It is challenging for investigators to maintain blinding, that is, the SEEDS and this substudy could only be single-blinded trials. Several measures will be taken to minimize bias. For instance, only PI has access to the allocation sequence, independent investigators screen the eligible participants, and blinded investigators assess the outcomes. The investigators collecting the data and the statisticians are allowed to unmask the allocation only after the completion of recruitment and closeness of the database.

### Blinding-emergency unblinding {17b}

None. The allocation of interventions will remain undisclosed to participants and their authorized surrogates.

## Data collection methods

### Data collection for both the main trial and the substudy {18a}

In the substudy, data are collected from patients’ electronic records, involving laboratory tests, imaging examinations, outpatient treatments, and telephone follow-up. Demographic data, including age, height, weight, gender, comorbidities, smoking, and regular drinking collected in the SEEDS main trial, are further collected into the substudy. Other relevant data from the main trial will be submitted to this substudy as well, including admission type (medical, surgical, or traumatic), location before admission (common ward, emergency room, operating room, or other ICU), APACHE II score, SOFA (baseline and daily score), daily CAM-ICU assessment, reason for intubation, duration of MV, daily total and maximum doses of study drugs, total dose of intraoperative anesthetics for patients after surgeries, vital signs, blood routine test, and liver and kidney functions.

### Additional data collection for substudy only

- PRE-DELIRIC [25] score; feature of delirium including subtype, CAM-S, time to first onset of delirium, and duration
- Daily arterial blood gas including pH, PaO_2_/FiO_2_ ratio, PaCO_2_, SaO_2_, lactate, and hematocrit
- Total dose of vasopressors and antipsychotics
- Total dose of glucocorticoids and anticholinergic agents (orally or intravenously)
- ICU and hospital LOS
- In-ICU mortality, mortality at 14 and 28 days; re-admission, accidental extubation, and re-intubation

### Retention and patient involvement statement {18b}

Family engagement and empowerment are essential components of delirium prevention. Personalized sedation strategies take the patient’s feelings as a priority. Therefore, patients and their families will be involved in the study process. We encourage family members to help participants recognize the benefits of study drugs during daily visits. This sedation protocol can facilitate the process of awakening and bring participants with more comforts, thereby fostering their confidence in receiving the study drugs. If participants drop out under the willingness of their own or surrogates, we will make efforts to gain their consents and preserve as many data as possible prior to the dropout. If possible, we will also record the duration of MV, LOS, and survival data. Participants who withdraw or drop out will not be followed up.

## Data management {19, 27 and 29}

Daily RASS and CPOT scores, CAM-ICU assessment, severity of delirium, treatment for delirium, and all AEs will be documented in the medical records as well as paper and electronic CRFs by two investigators to ensure accuracy. The paper CRFs will be secured by PI and then reviewed by the ethics committee. The electronic CRFs are password-protected files which can only be accessed by PI and investigator responsible for data collection. The password will be changed every month to ensure the privacy and confidentiality. After the last patient completes his or her issues in this study, all data will be locked without any further modification and subsequently provided to the statistician. The final dataset will be retained for 2 years.

## Statistical methods

### Primary outcome {20a}

The data about primary outcome, occurrence of delirium, are binary and qualitative. We will report this outcome as a point estimate of effect with 95% CIs using Clopper-Pearson method and analyze it using chi-square test. If any theoretical frequency is less than 5, the data will be analyzed after continuity correction. Fisher’s exact text will be performed if any theoretical frequency is further less than 1.

### Secondary outcomes {20a}

- Secondary outcomes, including delirium-free days, duration of delirium, dose of vasopressors and antipsychotics, and duration of mechanical ventilation, will be expressed as continuous data and analyzed by Student’s *t* test if showing satisfied normality and homogeneity of variance and otherwise by Mann-Whitney test
- Secondary outcomes, including subtype of delirium, occurrence of re-admission, re-intubation, and self-extubation, will be expressed as qualitative data and analyzed by chi-square test, as we do for the data about the primary outcome
- Time to first onset of delirium and survival time with 14 and 28 days will be presented as Kaplan-Meier curve and analyzed by log-rank test
- The severity of delirium will be assessed based on CAM-S score: none = 0, mild = 1, moderate = 2, severe = 3~7. The proportion of different levels of severity will be analyzed by chi-square test
- Two-tailed *P* value less than 0.05 will be regarded as statistically significant. All statistical tests will be run on SPSS 27.0 (IBM, USA)

### Additional analyses {20b}

Considering that one participant may experience recurrent delirium with increasing episodes, we will further use negative binomial regression to determine the association between the occurrence of delirium and the type of treatment, with the baseline characteristics as covariates. The consistency between treatment effects on the primary outcome will be analyzed in subgroups stratified according to age, gender, sepsis status, duration of mechanical ventilation, surgery status, and PRE-DELIRIC score before allocation.

### Analysis of population and missing data {20c}

Primary and secondary outcomes will be analyzed using an intention-to-treat approach. Participants will receive study drugs during their ICU stay, and their data will be analyzed according to their intervention, even if they withdraw from the trial or receive drugs of the other arm. Our analyses are based on the full analysis set to ensure the effect of randomization.

We expect that CAM-ICU assessment will be missed in few patients, because participants with light sedation can always cooperate with our simple commands. When assessments are unavailable (e.g., in the operating room or going out for examination), the mechanism of missing data should be missing at random (MAR). We will use the multiple imputation (MI) to impute the missing data. Sensitivity analysis will be performed by comparing the data set with or without imputation to ascertain the stability of data.

## Data monitoring

### Formal committee {21a}

None. No committee is needed to monitor the data in this substudy. For patients who need prolonged MV, sedation is a common treatment. In this substudy, only low-risk interventions are performed. The investigator responsible for data collection will be blinded to the allocation and not involved in the interventions. The whole process of data collection is independent from the sponsor. All the researchers in the study declare that they have no competing interests. The ethics committee will periodically review the data and recommend PI of continuation and modification of trials.

### Interim analysis {21b}

Not applicable. We decide not to conduct an interim analysis.

## Safety monitoring, adverse event reporting and harms {22}

As a part of the SEEDS trial, we keep alert to adverse events by monitoring the symptoms, vital signs, and laboratory test results. Throughout the administration of study drugs until extubation, the investigators will communicate closely with the clinical team to ensure the safety of the trial, and the safety monitoring investigators will remain blinded. During the period from extubation to ICU discharge, adverse events will be monitored daily. Vital signs will be recorded every hour by bedside nurses, and routine laboratory test will be performed every day. We anticipate that the following adverse events may occur: hypotension, hypertension, tachycardia, bradycardia, delayed awakening, allergy, drug-induced liver, or kidney injuries. A greater than 20% change from baseline heart rate and blood pressure will be considered as an adverse event. Liver injury is defined as ALT ≥ 5× upper limit of normal (ULN) or ALP ≥ 2× ULN or ALT ≥ 3× ULN combined with TBil ≥ 2× ULN, while kidney injury is defined as a new onset of kidney injury or the medication-induced worsening of an existing injury. PI will review the adverse event reports and determine the severity of the adverse events according to the Common Terminology Criteria for Adverse Events. Serious adverse events contain death, acute life-threatening condition, hospitalization or prolonged hospitalization, persistent and severe disability, and impairment of work ability. We will report all serious adverse events determined to be probable or definite based on the criteria in Table 4 to the ethics committee. Incidences of adverse events in both arms will be compared by safe analysis set.

**Table 4:** Criteria for determining the relationships between study drugs and adverse events

Items	Definite	Probable	Unlikely	Unrelated	Undecidable
Time to onset is logical	Yes	Yes	Yes	Yes	No
Type of response to the drug is known	Yes	Yes	Yes	No	No
Improvement after cessation of the drug	Yes	Yes	Yes/No	Yes/No	No
Reappear to unintentional re-exposure	Yes	No	No	No	No
Alternative causes	No	No	No	Yes	Yes

Incidence of adverse events will be calculated as the total number of events determined as *Definite* plus *Probable* plus *Undecidable* divide by all participants in the safe analysis set

## Auditing {23}

Before the initiation of the trial, the standard operating procedure will be established to assess the level of pain and sedation, to perform the daily sedation interruption, SBT, and extubation. During the trial, the ethics committee will conduct a comprehensive auditing every 3 months, and the frequency of auditing will be adjusted based on the pace of enrollment. The PI will carry out monthly conferences to audit the reports about ineligibility, dropouts, non-compliance, and cumulative adverse events. After the last patient completes his or her issues related to the study, the database will be locked. Final auditing will be conducted to determine whether the whole process sticks to the good clinical practice principle.

## Protocol amendments {25}

Once the protocol is approved by all investigators, the important components, such as study objectives, sample sizes, and patient population, will remain unchanged throughout the trial. If a major modification is necessary due to changes in government regulations, concerns about patient safety, or other reasons, PI will apply for formal amendments to the ethics committee. Modifications prior to the report are permissible if they aim to prevent emergency injuries. The amendments must be approved before implementation and then updated on ClinicalTrials.gov. Minor amendments which just exert a minimal impact on the trial will be documented in a memorandum.

## Dissemination policy {31a}

The results of this substudy will be published on peer-reviewed journal or presented at academic meetings.

## Discussion

For patients who need prolonged MV in the ICU, sedatives should be suited to achieve satisfactory sedative effects, an easily adjustable depth of sedation, a minimal impact on circulatory and respiratory function, and a low incidence of delirium. Evidence is available to show that dexmedetomidine can reduce the risk of delirium [11, 26, 27]. However, its application is limited by such factors as delayed onset of sedation [28], relatively mild sedative effects, and evident impacts on hemodynamics. A sedation in MV patients can only be called satisfactory in the presence of stable hemodynamics. Moreover, the combination with benzodiazepines, such as midazolam, counteracts the anti-delirium effect of dexmedetomidine, while the combination with propofol may aggravate hypotension, thus necessitating the use of vasoactive agents. Therefore, there is an urgent need for a new combination regimen of dexmedetomidine. The purpose of the SEEDS main trial is to evaluate the safety and efficacy of the combination of esketamine and dexmedetomidine for MV patients. This substudy, as a part of the SEEDS, will provide evidence about whether this combination can reduce the risk of delirium and improve the short-term prognosis of critically ill patients.

This regimen has been applied throughout the perioperative period, from induction, maintenance, to postoperative analgesia, but its effects on the risk of delirium are controversial. Lu et al. [24] have used this regimen to induce anesthesia in children, suggesting that the incidence of emergence agitation and the Pediatric Anesthesia Emergence Delirium score in the combination arm are significantly lower than those in the esketamine-alone and dexmedetomidine-alone arms. In a study by Huang et al. [13], this regimen is used for intraoperative anesthesia in modified radical mastectomy for breast cancer; esketamine is highly (0.5 mg/kg loading and 4 μg/kg/min infusion) and lowly (0.5 mg/kg loading and 2 μg/kg/min infusion) dosed; the results reveal no significant difference in the incidence of postoperative agitation within 30 min after surgery among the dexmedetomidine-alone arm, low-dose combination arm, and high-dose combination arm. Zhang et al. [29] have used a mini-dose (average infusion rate of 5.5 μg/kg/h for esketamine and 0.02 μg/kg/h for dexmedetomidine) combination regimen for supplemental patient-controlled analgesia after scoliosis surgery; no patients undergo delirium in the combination arm and the placebo arm within 5 days after surgery, making it difficult to determining the differences between the two arms. Although the use of sedatives and analgesics in the ICU is quite different from that in clinical anesthesia, we still encourage the combination of both drugs for MV patients.

If the combination does reduce the risk of delirium, we will further explore whether this benefit can be translated into as an increase in delirium-free days, a reduction in severity, a delay of onset, or a shortening of duration. Moreover, the decrease in the odds of delirium may be achieved in several ways. First, both esketamine and dexmedetomidine have adjuvant analgesic effects [30, 31]. Esketamine mainly exerts analgesic effects by blocking NMDA receptors and also relieves opioid-induced hyperalgesia [32]. Dexmedetomidine excites α_2_ adrenergic receptors to inhibit the release of excitatory neurotransmitters and hyperpolarize cells, thereby inhibiting pain signaling [33]. Both can reduce the use of opioid analgesia through eliciting multimodal analgesia. In addition, the antidepressant effect of esketamine may reduce the use of antipsychotic drugs in the ICU. Second, in this combination, both drugs can counteract each other's side effects to reduce the use of vasoactive drugs, especially for patients transferred to the ICU after major surgeries, thus repressing the occurrence of delirium. Third, the combination can place the patient at a stable level of light sedation, reducing the risk of accidental extubation and reintubation. We have exploratively designed several secondary outcomes to verify the above hypotheses. Furthermore, to simulate the actual application of sedatives in the ICU, we will carry out subgroup analyses to evaluate the effects of this combination in some specific populations, such as elderly patients, septic patients, patients with extreme prolonged MV, post-surgery patients, and patients with a high risk of delirium prior to the enrollment.

So far, no studies have appeared to confirm whether the combination of esketamine and dexmedetomidine can further reduce the risk of delirium for MV patients in the ICU, a question expected to be resolved in this substudy. However, there are some limitations in this substudy. First, since patients with craniocerebral injury or severe acute respiratory distress syndrome often have difficulties in cooperating with delirium assessment or require continuous deep sedation after being transferred to the ICU, they will be excluded from our study. So, it is difficult to evaluate the effect of this regimen on these patients. Second, there is still a lack of high-quality evidence on the effects of esketamine combined with dexmedetomidine on delirium in MV patients, which will be initially explored in this substudy. Therefore, this trial is a small-sample, single-center study, and multicenter studies with more samples are still needed in the future. Third, this study reflects the combined effect of esketamine and dexmedetomidine on delirium. Subsequent factorial trials are needed to determine the effect of esketamine on delirium among MV patients in the ICU. This combination regimen still has other benefits worth exploring.

## Trial status

The current protocol containing substudy is the version 3, which was completed on 08/01/2023. Recruitment for the substudy began in August 2023, and it is expected to be finished by December 2024.

### Supplementary Information

Additional file 1. SPIRIT 2013 Checklist: Recommended items to address in a clinical trial protocol and related documents. 

## Data Availability

After publication, the full protocol of the substudy and main trial, randomization sequence, block size, seed number, and statistical code will be available from the corresponding author on request. The study results will be submitted to ClinicalTrials.gov within 1 year of the completion date of the primary outcome. The final data are available from the corresponding author on reasonable request.

## Abbreviations

ICU: Intensive care unit
MV: Mechanical ventilation/mechanically ventilated
LOS: Length of stay
NMDA: N-methyl-d-aspartate
CAM-ICU: The Confusion Assessment Method for ICU
RASS: The Richmond Agitation and Sedation Scale
CPOT: The Critical-care Pain Observation Tool
CRF: Case report form
SBT: Spontaneous breathing trial
PI: The principal investigator
APACHE II: Acute Physiology and Chronic Health Evaluation II
SOFA: Sequential Organ Failure Assessment
ULN: Upper limit of normal

## References

[1] Wilson JE, Mart MF, Cunningham C, Shehabi Y, Girard TD, MacLullich AMJ (2020). Delirium. Nat Rev Dis Primers..

[2] Jayaswal AK, Sampath H, Soohinda G, Dutta S (2019). Delirium in medical intensive care units: incidence, subtypes, risk factors, and outcome. Indian J Psychiatry.

[3] Pandharipande P, Cotton BA, Shintani A, Thompson J, Pun BT, Morris JA (2008). Prevalence and risk factors for development of delirium in surgical and trauma intensive care unit patients. J Trauma.

[4] Chaiwat O, Chanidnuan M, Pancharoen W, Vijitmala K, Danpornprasert P, Toadithep P (2019). Postoperative delirium in critically ill surgical patients: incidence, risk factors, and predictive scores. BMC Anesthesiol.

[5] Stollings JL, Kotfis K, Chanques G, Pun BT, Pandharipande PP, Ely EW (2021). Delirium in critical illness: clinical manifestations, outcomes, and management. Intensive Care Med.

[6] Ely EW, Shintani A, Truman B, Speroff T, Gordon SM, Harrell FE (2004). Delirium as a predictor of mortality in mechanically ventilated patients in the intensive care unit. JAMA.

[7] Gitti N, Renzi S, Marchesi M, Bertoni M, Lobo FA, Rasulo FA (2022). Seeking the light in intensive care unit sedation: the optimal sedation strategy for critically ill patients. Front Med (Lausanne).

[8] Duprey MS, Dijkstra-Kersten SMA, Zaal IJ, Briesacher BA, Saczynski JS, Griffith JL (2021). Opioid use increases the risk of delirium in critically ill adults independently of pain. Am J Respir Crit Care Med.

[9] Riker RR, Shehabi Y, Bokesch PM, Ceraso D, Wisemandle W, Koura F (2009). Dexmedetomidine vs midazolam for sedation of critically ill patients: a randomized trial. JAMA.

[10] Devlin JW, Skrobik Y, Gélinas C, Needham DM, Slooter AJC, Pandharipande PP (2018). Clinical practice guidelines for the prevention and management of pain, agitation/sedation, delirium, immobility, and sleep disruption in adult patients in the ICU. Crit Care Med.

[11] Lewis K, Alshamsi F, Carayannopoulos KL, Granholm A, Piticaru J, Al Duhailib Z (2022). Dexmedetomidine vs other sedatives in critically ill mechanically ventilated adults: a systematic review and meta-analysis of randomized trials. Intensive Care Med.

[12] Jakob SM, Ruokonen E, Grounds RM, Sarapohja T, Garratt C, Pocock SJ (2012). Dexmedetomidine vs midazolam or propofol for sedation during prolonged mechanical ventilation: two randomized controlled trials. JAMA.

[13] Huang Z, Liu N, Hu S, Ju X, Xu S, Wang S (2023). Effect of dexmedetomidine and two different doses of esketamine combined infusion on the quality of recovery in patients undergoing modified radical mastectomy for breast cancer - a randomised controlled study. Drug Des Devel Ther.

[14] Kim JG, Lee HB, Jeon SB (2019). Combination of dexmedetomidine and ketamine for magnetic resonance imaging sedation. Front Neurol.

[15] Frestadius A, Grehn F, Kildal M, Huss F, Fredén F (2022). Intranasal dexmedetomidine and rectal ketamine for young children undergoing burn wound procedures. Burns.

[16] Lin Z, Li S, Zhou Y, Lu X, Yang B, Yu Z (2023). A comparative study of esketamine-dexmedetomidine and sufentanil-dexmedetomidine for sedation and analgesia in lung tumor percutaneous radiofrequency ablation (PRFA): a randomized double-blind clinical trial. BMC Anesthesiol.

[17] Lian X, Lin Y, Luo T, Jing Y, Yuan H, Guo Y (2023). Efficacy and safety of esketamine for sedation among patients undergoing gastrointestinal endoscopy: a systematic review and meta-analysis. BMC Anesthesiol.

[18] Trimmel H, Helbok R, Staudinger T, Jaksch W, Messerer B, Schöchl H (2018). S(+)-ketamine : current trends in emergency and intensive care medicine. Wien Klin Wochenschr.

[19] Lu Y, Yin G, Jin C, Gu K, Bao D, Xu W (2024). The application value of esketamine and dexmedetomidine in preventing postoperative delirium and hyperalgesia in elderly patients with thoracic anesthesia. Altern Ther Health Med.

[20] Ma J, Wang F, Wang J, Wang P, Dou X, Yao S (2023). The effect of low-dose esketamine on postoperative neurocognitive dysfunction in elderly patients undergoing general anesthesia for gastrointestinal tumors: a randomized controlled trial. Drug Des Devel Ther.

[21] Chen Y, Ru F, Ye Q, Wu X, Hu X, Zhang Y (2023). Effect of S-ketamine administered at the end of anesthesia on emergence delirium in preschool children undergoing tonsillectomy and/or adenoidectomy. Front Pharmacol.

[22] Wang C, Wu Y, Yue P, Ely EW, Huang J, Yang X (2013). Delirium assessment using Confusion Assessment Method for the Intensive Care Unit in Chinese critically ill patients. J Crit Care.

[23] Inouye SK, Kosar CM, Tommet D, Schmitt EM, Puelle MR, Saczynski JS (2014). The CAM-S: development and validation of a new scoring system for delirium severity in 2 cohorts. Ann Intern Med.

[24] Lu X, Tang L, Lan H, Li C, Lin H (2021). A comparison of intranasal dexmedetomidine, esketamine or a dexmedetomidine-esketamine combination for induction of anaesthesia in children: a randomized controlled double-blind trial. Front Pharmacol.

[25] van den Boogaard M, Pickkers P, Slooter AJ, Kuiper MA, Spronk PE, van der Voort PH (2012). Development and validation of PRE-DELIRIC (PREdiction of DELIRium in ICu patients) delirium prediction model for intensive care patients: observational multicentre study. BMJ.

[26] Burry LD, Cheng W, Williamson DR, Adhikari NK, Egerod I, Kanji S (2021). Pharmacological and non-pharmacological interventions to prevent delirium in critically ill patients: a systematic review and network meta-analysis. Intensive Care Med.

[27] Ng KT, Shubash CJ, Chong JS (2019). The effect of dexmedetomidine on delirium and agitation in patients in intensive care: systematic review and meta-analysis with trial sequential analysis. Anaesthesia.

[28] Kim JY, Kim KN, Kim DW, Lim HJ, Lee BS (2021). Effects of dexmedetomidine sedation for magnetic resonance imaging in children: a systematic review and meta-analysis. J Anesth.

[29] Zhang Y, Cui F, Ma JH, Wang DX (2023). Mini-dose esketamine-dexmedetomidine combination to supplement analgesia for patients after scoliosis correction surgery: a double-blind randomised trial. Br J Anaesth.

[30] Xu LL, Wang C, Deng CM, Dai SB, Zhou Q, Peng YB (2023). Efficacy and safety of esketamine for supplemental analgesia during elective cesarean delivery: a randomized clinical trial. JAMA Netw Open.

[31] Gao Y, Deng X, Yuan H, Leng Y, Zhang T, Xu X (2018). Patient-controlled intravenous analgesia with combination of dexmedetomidine and sufentanil on patients after abdominal operation: a prospective, randomized, controlled, blinded, multicenter clinical study. Clin J Pain.

[32] Colvin LA, Bull F, Hales TG (2019). Perioperative opioid analgesia-when is enough too much? A review of opioid-induced tolerance and hyperalgesia. Lancet.

[33] Guo X, Xue Y, Ji W, Liang J, Qingshi Z (2022). Effects of administration of α(2) adrenergic receptor agonist into psoas major muscle on inflammatory pain induced by injection of complete Freund's adjuvant in rats. Mol Pain.

